# Editorial: Unraveling the complexity of sensory space perception

**DOI:** 10.3389/fpsyg.2025.1721743

**Published:** 2025-11-12

**Authors:** Givago Silva Souza, Francisco dos Santos Cardoso, Javier Enrique Santillan, José Fernando Barraza, José Aparecido Da Silva

**Affiliations:** 1 Núcleo de Medicina Tropical, Universidade Federal do Pará, Belém, Brazil; 2 Instituto de Ciências Biológicas, Universidade Federal do Pará, Belém, Brazil; 3 Universidade de Trás-os-Montes e Alto Douro, Vila Real, Portugal; 4 Universidad Nacional de Tucumán, San Miguel de Tucumán, Argentina; 5 Psychobiology Unit, University of São Paulo, Ribeirão Preto, Brazil

**Keywords:** spatial cognition, perception, sensory system, vestibular system, visual system, proprioception, hearing, haptic (tactile) perception

Environmental sensing refers to the process by which organisms extract information from their surroundings to construct representations that guide behavior (Swanson, 2011). Sensory recognition and the perceptual construction of such information are inherently complex, given the multiplicity of mechanisms involved in each sensory system (Kaas, 1989). Spatial perception is fundamental to survival, as it enables the identification of food, the location of shelters, and the detection of predators, allowing organisms to rapidly adjust their actions to avoid risks.

At the functional level, spatial perception supports navigation through the environment (Loomis et al., 1992; Yamamoto and Philbeck, 2025), distance estimation (Santillán and Barraza, 2019), balance control (Corrêa et al., 2023), emotional expression (Cardoso et al., 2021), and the coordination of complex movements (Angelaki et al., 2025). Whether it is a bat in flight, a fish swimming against the current, or a human walking across uneven terrain, all rely on spatial integration to efficiently and safely explore their surroundings. On a broader scale, spatial recognition underpins social and cognitive life (Proulx et al., 2016). In social animals, spatial signals can help distinguish among different social contexts (Dorfman and Eilam, 2021). In humans, this ability is closely linked to abstract reasoning (Harris, 2023).

Human spatial perception arises from the integration of multiple sensory systems (Bremmer, 2011). No single sense, in isolation, can provide all the information required for an individual to determine their position in the environment, orient themselves relative to objects, and plan appropriate motor or cognitive actions. It is the dynamic combination of sensory inputs that enables the construction of a coherent representation of space. Although this strategy may introduce redundancy, since sensory modalities can convey overlapping information, the engagement of multiple senses in building perceptual representations reduces the likelihood of processing errors (Ernst and Bülthoff, 2004). The relative weight of each modality varies according to context and the reliability of the available information (Burns and Blohm, 2010). This flexibility ensures the robustness of spatial perception across a wide range of contexts, from everyday life to extreme conditions, such as virtual environments and microgravity (Bogon et al., 2024; Glasauer and Mittelstaedt, 1998).

The present Research Topic, entitled *Unraveling the Complexity of Sensory Space Perception*, was designed to gather contributions that expand our understanding of how sensory systems process spatial information and how novel methods can facilitate the investigation of these processes. Six articles were accepted, addressing different sensory modalities: the vestibular system (Zhang et al., 2025; Gerb et al., 2024), vision (Takeichi et al., 2025), proprioception (Almeida et al., 2025), audition (Metcalfe and Harris, 2025), and visuo-haptic integration (Fischer et al., 2025).

Gerb et al. (2024) investigated the relationship between subjective discomfort in spatial orientation and performance on objective real-world navigation tasks, along with its association with cognitive function in individuals with normal vestibular function. Their findings suggest that the subjective sensation of spatial discomfort may serve as a valuable clinical marker, correlating with both behavioral performance and cognitive decline. The study emphasizes that spatial orientation assessment should integrate subjective and objective measures.

Zhang et al. (2025) examined the optimal rotational speed for the unilateral centrifugation subjective visual vertical (UC-SVV) test, in which participants adjust a luminous line to what they perceive as the true vertical in the absence of external references. This test specifically targets otolithic vestibular function. The study provides practical parameters for standardizing the UC-SVV test in clinical contexts, indicating that rotational speeds of 180°/s (or 240°/s, if tolerated) enhance the test's sensitivity and reliability in detecting vestibular dysfunctions.

Takeichi et al. (2025) tested whether the human visual system can extract information about physical properties of fluids, such as viscosity, from non-rigid motion. Using the pseudo-flow technique, based on tracking image gradient vectors, they demonstrated that vision can infer the physical properties of non-rigid structures from local motion, advancing our understanding of how the visual system encodes motion and structure in the environment.

Almeida et al. (2025) compared joint position sense measures obtained with inertial sensors embedded in devices of different masses: a heavier smartphone and an ultralight sensor. In elbow repositioning tasks, they observed a systematic bias associated with the smartphone, which also showed moderate-to-good test–retest reliability. By contrast, the ultralight sensor exhibited poor-to-moderate reliability. The study concludes that, while both devices are suitable for assessing proprioception, device mass systematically affects outcomes and must be considered in clinical and experimental applications.

Metcalfe and Harris (2025) examined how prior knowledge influences the perception of vocal elements in MIDI stimuli, even in the absence of an actual voice. The authors found that a vague illusion of vocal presence can occur without prior learning, but that only previous familiarity with the music enables the precise perception of words. These findings underscore the fact that auditory perception is not passive but rather emerges from the interplay between bottom-up sensory cues and top-down cognitive expectations.

Fischer et al. (2025) characterized precision and accuracy in target localization tasks performed under visual, haptic, and combined visuo-haptic conditions. Their results reveal that integration does not always follow ideal models of sensory combination and that structural differences between visual and haptic maps shape spatial perception. These findings hold important implications for the design of human–machine interfaces with haptic feedback and for applications in virtual reality and robotics.

In conclusion, the original objective of this Research Topic was successfully achieved. The studies presented here reveal new aspects of the complexity of sensory processing and its relationship with the spatial domain. As emphasized in the call for this Research Topic, we remain confident that advances from diverse fields, including psychology, neuroscience, and computer science, will continue to elucidate environmental information processing, with implications not only for the clinical assessment of the nervous system but also for the development and application of current and future technologies in the service of humanity.

## References

[B1] Almeida J. R. Monteiro L. C. P. de Souza P. H. C. Cabral A. S. Belgamo A. de Athayde Costa e Silva A. . (2025). Comparison of joint position sense measured by inertial sensors embedded in portable digital devices with different masses. Front. Neurosci. 19:1561241. doi: 10.3389/fnins.2025.1561241 40433496 PMC12106428

[B2] Angelaki D. Benson B. Benson J. Birman D. Bonacchi N. Bougrova K. . (2025). A brain-wide map of neural activity during complex behaviour. Nature 645, 177–191. doi: 10.1038/s41586-025-09235-0 40903598 PMC12408349

[B3] Bogon J. Jagorska C. Steinecker I. Riemer M. (2024). Age-related changes in time perception: effects of immersive virtual reality and spatial location of stimuli. Acta Psychol. 249:104460. doi: 10.1016/j.actpsy.2024.104460 39126911

[B4] Bremmer F. (2011). Multisensory space: From eye-movements to self-motion. J. Physiol. 589, 815–823. doi: 10.1113/jphysiol.2010.195537 20921203 PMC3060361

[B5] Burns J. K. Blohm G. (2010). Multi-sensory weights depend on contextual noise in reference frame transformations. Front. Hum. Neurosci. 4:221. doi: 10.3389/fnhum.2010.00221 21165177 PMC3002464

[B6] Cardoso F. S. Teixeira L. E. P. P. de Freitas R. L. Abad A. Campos L. A. M. Da Silva J. A. . (2021). Peritraumatic distress caused by the COVID-19 pandemic: comparison between genders and countries—Brazil and Portugal. Mankind. Q. 62, 239–254. doi: 10.46469/mq.2021.62.2.2

[B7] Corrêa B. D. C. Santos E. G. R. Belgamo A. Pinto G. H. L. Xavier S. S. Silva C. C. . (2023). Smartphone-based evaluation of static balance and mobility in long-lasting COVID-19 patients. Front. Neurol. 14:1277408. doi: 10.3389/fneur.2023.1277408 38148981 PMC10750373

[B8] Dorfman A. Eilam D. (2021). Social spatial cognition: social distance dynamics as an identifier of social interactions. Anim. Cogn. 24, 407–418. doi: 10.1007/s10071-020-01441-9 33048261

[B9] Ernst M. O. Bülthoff H. H. (2004). Merging the senses into a robust percept. Trends Cogn. Sci. 8, 162–169. doi: 10.1016/j.tics.2004.02.002 15050512

[B10] Fischer M. Saetti U. Godfroy-Cooper M. Fischer D. (2025). Characterization of 2D precision and accuracy for combined visual-haptic localization. Front. Neurosci. 19:1528601. doi: 10.3389/fnins.2025.1528601 40143850 PMC11936952

[B11] Gerb J. Oertle V. Becker-Bense S. Brandt T. Dieterich M. (2024). Subjective spatial orientation discomfort is associated with decreased real-world spatial performance and lower cognitive function. Front. Neurosci. 18:1481653. doi: 10.3389/fnins.2024.1481653 39605790 PMC11599218

[B12] Glasauer S. Mittelstaedt H. (1998). Perception of spatial orientation in microgravity. Brain Res. Brain Res. Rev. 28, 185–193. doi: 10.1016/S0165-0173(98)00038-1 9795210

[B13] Harris D. (2023). Spatial reasoning in context: Bridging cognitive and educational perspectives of spatial-mathematics relations. Front. Educ. 8:1302099. doi: 10.3389/feduc.2023.1302099

[B14] Kaas J. H. (1989). The evolution of complex sensory systems in mammals. J. Exp. Biol. 146, 165–176. doi: 10.1242/jeb.146.1.165 2689560

[B15] Loomis J. M. Da Silva J. A. Fujita N. Fukusima S. S. (1992). Visual space perception and visually directed action. J. Exp. Psychol. Hum. Percept. Perform. 18, 906–21. doi: 10.1037/0096-1523.18.4.906 1431754

[B16] Metcalfe S. Harris J. A. (2025). Prior knowledge yields more precise perception of vocal elements in MIDI-converted music. Front. Psychol. 16:1565292. doi: 10.3389/fpsyg.2025.1565292 41099020 PMC12518094

[B17] Proulx M. J. Todorov O. S. Taylor Aiken A. de Sousa A. A. (2016). Where am I? Who am I? The relation between spatial cognition, social cognition and individual differences in the built environment. Front. Psychol. 7:64. doi: 10.3389/fpsyg.2016.00554 26903893 PMC4749931

[B18] Santillán J. E. Barraza J. F. (2019). Distance perception during self-movement. Hum. Mov. Sci. 67:102496. doi: 10.1016/j.humov.2019.102496 31301557

[B19] Swanson L. W. (2011). “The sensory system: inputs from environment and body,” in Brain Architecture: Understanding the Basic Plan, 2nd Edn. Oxford: Oxford Academic.

[B20] Takeichi H. Suzuki W. Yamashita W. Hiyama A. (2025). Perception of nonrigid structures from motion using tracking image gradient vectors. Front. Psychol. 16:1586648. doi: 10.3389/fpsyg.2025.1586648 40934047 PMC12418981

[B21] Yamamoto N. Philbeck J. W. (2025). What visually directed action reveals about perception of ambulatory space. J. Exp. Psychol. Hum. Percept. Perform. 51, 1315–1318. doi: 10.1037/xhp0001302 40991785

[B22] Zhang X. Deng Q. Huang X. Wen C. Wang W. Chen T. . (2025). Clinical study on the optimal rotational stimulation for the unilateral centrifugation subjective visual vertical test. Front. Neurosci. 19:1529572. doi: 10.3389/fnins.2025.1529572 40035062 PMC11872882

